# The correlation of deep learning-based CAD-RADS evaluated by coronary computed tomography angiography with breast arterial calcification on mammography

**DOI:** 10.1038/s41598-020-68378-4

**Published:** 2020-07-13

**Authors:** Zengfa Huang, Jianwei Xiao, Yuanliang Xie, Yun Hu, Shutong Zhang, Xiang Li, Zheng Wang, Zuoqin Li, Xiang Wang

**Affiliations:** Department of Radiology, The Central Hospital of Wuhan, Tongji Medical College, Huazhong University of Science and Technology, 26 Shengli Avenue, Jiangan, Wuhan, 430014 Hubei China

**Keywords:** Cardiovascular diseases, Cancer screening

## Abstract

This study sought to evaluate the association of breast arterial calcification (BAC) on breast screening mammography with the Coronary Artery Disease-Reporting and Data System (CAD-RADS) based on Deep Learning-coronary computed tomography angiography (CCTA). This prospective single institution study included asymptomatic women over 40 who underwent CCTA and breast cancer screening mammography between July 2018 and April 2019. CAD-RADS was scored based on Deep Learning (DL). Mammograms were assessed visually for the presence of BAC. A total of 213 patients were included in the analysis. In comparison to the low CAD-RADS (CAD-RADS < 3) group, the high CAD-RADS (CAD-RADS ≥ 3) group, more often had a history of hypertension (*P* = 0.036), diabetes (*P* = 0.017), and chronic kidney disease (*P* = 0.006). They also had a significantly higher level of LDL-C (*P* = 0.024), while HDL-C was lower than in the low CAD-RADS group (*P* = 0.003). BAC was also significantly higher in the high CAD-RADS group (*P* = 0.002). In multivariate analysis, the presence of BAC [odd ratio (OR) 10.22, 95% CI 2.86–36.49, *P* < 0.001] maintained a significant associations with CAD-RADS after adjustment by meaningful variable. The same tendency was also found after adjustment by all covariates. There was a significant correlation between the severities of CAD detected by DL based CCTA and BAC in women undergoing breast screening mammography. BAC may be used as an additional diagnostic tool to predict the severity of CAD in this population.

## Introduction

Cardiovascular disease (CVD) is now the leading cause of mortality and morbidity worldwide^1,2^. China is undergoing a rapid epidemiological transition with particular implications for the growth of cardiovascular disease with an estimate of 290 million affected individuals^3–5^. Meanwhile breast cancer affects as many millions of women as CVD and women are commonly screened for breast cancer using mammography. It is reported that 47.5% of women aged 40 to 49 and 57.2% of women between 50 and 74 had mammograms in 2011^6^. However, there is no routine screening for coronary artery disease (CAD). Moreover, compared to men, women have been shown to have worse outcome when CAD is confirmed^7,8^.

A robust association has been observed between the presence of breast artery calcifications (BAC) and CVD since the association was first reported by Van Noord et al*.* in 1996^9^. A meta-analysis identified the prevalence of BAC as 12.7% by screening mammography screening^10^. However, this association between BAC and CAD has been reported only in the US and in other western countries. No evidence exists in the Chinese population link BAC to the Coronary Artery Disease-Reporting and Data System (CAD-RADS) in coronary CT angiography (CCTA).

Deep learning (DL) has been demonstrated the potential clinical application value in radiology^11^. Specifically, the preliminary work of using DL methods to evaluate CAD has been reported recently^12^. However, no research has showed the application of these techniques implemented in CAD-RADS. We aim to develop a DL algorithm for accuracy assessment of CAD and then, to evaluate the association between BAC on breast screening mammography and CAD-RADS grades based on DL-CCTA in women.

## Results

Baseline characteristics of the 213 study participants are shown in Table 1 (mean age: 58 years; range from 40 to 85 years). BAC was found in 22 (10.3%) of the women, while 23 of them were classified as CAD-RADS ≥ 3.

**Table 1:** Baseline characteristics of patients (n = 213).

Age	58 ± 8.6
**Medical history**	
Chest tightness	91 (42.7)
Chest pain	45 (21.1)
Myocardial infarction	1 (0.5)
Angina	25 (11.7)
Hypertension	94 (44.1)
Diabetes	42 (19.7)
CKD	25 (11.7)
TG	1.59 ± 0.88
TC	5.04 ± 0.99
HDL-C	1.37 ± 0.33
LDL-C	3.11 ± 0.83
Apo-A	1.41 ± 0.25
Apo-B	0.93 ± 0.2
FFA	0.53 ± 0.26
BUN	5.02 ± 1.99
CR	54 ± 15.72

*CKD*  Chronic kidney disease, *TG*  triglyceride, *TC*  total cholesterol, *HDL-C* High density lipoprotein-C, *LDL-C*  Low density lipoprotein-C, *Apo-A*  Apolipoprotein A, *Apo-B* Apolipoprotein B, *FFA*  Free fatty acids, *BUN*  Blood urea nitrogen, *CR*  Creatinine.

Univariate analysis showed that hypertension (OR 2.63, 95% CI 1.07–6.5, *P* = 0.036), diabetes (OR 3.06, 95% CI 1.22–7.66, *P* = 0.017), chronic kidney disease (OR 4.18, 95% CI 1.52–11.50, *P* = 0.006), high density lipoprotein-C (HDL-C; OR 0.23, 95% CI 0.09–0.62, *P* = 0.003), low density lipoprotein-C (LDL-C; OR 2.79, 95% CI 1.15–6.79, *P* = 0.024) and the presence of BAC (OR 5.1, 95% CI 1.82–14.34, *P* = 0.002) were all significant predictors of CAD-RADS ≥ 3 (Table 2).

**Table 2:** Univariate analysis to determine factors associated with CAD-RADS.

	OR	95% CI	*P* value
Age	0.99	0.94–1.04	0.673
Chest tightness	1.04	0.43–2.48	0.938
Chest pain	2.2	0.87–5.56	0.096
Angina	1.69	0.53–5.46	0.377
Hypertension	2.63	1.07–6.5	0.036
Diabetes	3.06	1.22–7.66	0.017
CKD	4.18	1.52–11.50	0.006
TG	1.56	0.66–3.82	0.302
TC	0.71	0.29–1.73	0.456
HDL-C	0.23	0.09–0.62	0.003
LDL-C	2.79	1.15–6.79	0.024
Apo-A	0.77	0.25–2.37	0.643
Apo-B	0.63	0.22–1.76	0.386
BUN	0.89	0.68–1.17	0.405
CR	0.98	0.97–1.03	0.869
BAC	5.1	1.82–14.34	0.002

*CKD* Chronic kidney disease, *TG* triglyceride, *TC* total cholesterol, *HDL-C* High density lipoprotein-C, *LDL-C* Low density lipoprotein-C, *Apo-A* Apolipoprotein A, *Apo-B* Apolipoprotein B, *BUN* Blood urea nitrogen, *CR* Creatinine, *BAC* Breast arterial calcification.

In multivariate analysis (Table 3), chronic kidney disease (OR 5.33, 95% CI 1.56–18.23, *P* = 0.008), HDL-C (OR 0.24, 95% CI 0.58–0.76, *P* = 0.015), LDL-C (OR 3.55, 95% CI 1.24–10.16, *P* = 0.018) and the presence of BAC (OR 10.22, 95% CI 2.86–36.49, *P* < 0.001) maintained significant associations with CAD-RADS after adjustment by meaningful variable, whereas hypertension and diabetes did not. The same tendency was also found after adjustment by all covariates. BAC offers a high negative predictive value of 91.6% (95% CI 86.8–95.1) and moderate positive predictive value of 68.2% (95% CI 45.1–86.1).

**Table 3:** Multivariate analysis associated with CAD-RADS.

	Model 1			Model 2		
	OR	95% CI	*P* value	OR	95% CI	*P* value
Hypertension	2.72	0.97–7.6	0.056	2.18	0.72–6.62	0.171
Diabetes	2.44	0.85–7.01	0.099	2.32	0.67–8.03	0.185
CKD	5.33	1.56–18.23	0.008	6.04	1.50–24.30	0.011
HDL-C	0.24	0.08–0.76	0.015	0.27	0.08–0.93	0.037
LDL-C	3.55	1.24–10.16	0.018	8.42	1.98–35.86	0.004
BAC	10.22	2.86–36.49	< 0.001	23.85	4.99–113.96	< 0.001

*CKD* Chronic kidney disease, *HDL-C* High density lipoprotein-C, *LDL-C* Low density lipoprotein-C, *BAC* Breast arterial calcification. Model 1, adjusted by hypertension, diabetes, CKD, HDL-C, LDL-C; Model 2, adjusted by age, chest tightness, chest pain, angina, hypertension, diabetes, CKD, TG, TC, HDL-C, LDL-C, Apo-A, Apo-B, BUN, CR.

## Discussion

The present study first demonstrates the correlation between BAC on breast screening mammography and severity of CAD as defined by the DL-based CAD-RADS. In addition, BAC predicted a CAD-RADS score of 3 or greater in women undergoing breast screening mammography.

Previous studies have demonstrated that age is the strongest determinant of survival, whereas sex has conflicting and less significant effects on risk^13^. However, in the present study, age was not associated with DL-based CAD-RADS grades, partly because all the included patients were women and the lower age limit for the population was 40. Traditional cardiovascular risk factors such as hypertension and diabetes^14^, were significantly associated with DL-based CAD-RADS grade ≥ 3 in univariate analysis. However, there was no significant difference in multivariate analysis. One possible reason for this difference may be the small sample size. Chronic kidney disease (CKD) is the most important noncardiac condition associated with CAD which has been found to influence prognosis^15^. In multivariate analysis, CKD was detected as an independent risk factor for DL-based CAD-RADS grade ≥ 3, which was similar to a previous study^16^. BAC assessed by mammography was evaluated as a potential risk stratification tool and surrogate marker of CAD^17^. The prevalence of BAC using mammography varies widely among published studies which range from 10 to 12%^10,18^. These differences may be due to the heterogeneity of the populations. In the present study, the prevalence of detected BAC was 10.3%. However, the prevalence of BAC has increased over time with technical advancements in mammography^19^.

Some small studies have suggested that there is no significant association between BAC and CAD^20–22^. However, multiple studies, including some prospective studies, have demonstrated a strong association between BAC and CAD or CVD, independent of other known CAD risk factors^9,23–26^. The differences between studies may be due to variation in the way the primary outcome of CAD is defined, such as self-report, coronary artery calcification on computed tomography, CCTA or chart diagnosis using International Classification of Diseases codes. Most studies refer either to the absence or presence of BAC or to a grading system (1–4 or 0–3) or an alternative system enumerating calcified arteries. The calcium score, especially the Agaston Score, has been increasingly used as an indicator of CAD in CCTA^23^. However, the correlation between BAC and CAD-RADS grades was scarcely reported. The main purpose for the CAD-RADS calcification system proposed by the American College of Radiology was to standardise CCTA reporting and decrease the potential discrepancy between physicians in the reporting of stenosis. In the present study, our data clearly showed that BAC is associated with CAD-RADS grade ≥ 3. The mechanism of this may be complex and is incompletely understood. It is important to realise that the pathogenesis of BAC and CAD are separate and their locations are different. Unlike the intimal location of CAD calcification, BAC is manifested as calcific sclerosis which is medial in location^27^. A recent editorial revealed that these mechanisms of calcification have modifiable risk factors in common with breast cancer which was the initial reason for the mammogram^28^. This indicates that screening mammography could be a potential platform for reducing the risk of both breast cancer and CAD by identifying common risk factors for the separate pathogenic processes.

CAD-RADS can now guide clinical decision-making using CCTA and may play a significant role in connecting lesion detections with optimal patient care. The recent multinational CONFIRM study revealed that standardized CCTA reports incorporated with CAD-RADS might promote the development of evidence-based care post-CCTA^29^. BAC predicted a CAD-RADS grade ≥ 3 in the present study. This was important because a CAD-RADS grade of 3 or greater suggests consideration of functional evaluation and anti-ischemic or preventative drugs. A previous small cohort study found a positive correlation between BAC detected by screening mammography on symptomatic women and CAD-RADS score^30^, which was consistent with our findings. However, our findings are more widely applicable as the included patients in the present study were not just those with chest pain, but all women undergoing mammography. Moreover, the CAD-RADS scoring system used in the present study was based on DL, making determination of CAD-RADS grade by measuring stenosis degree faster, more objective and repeatable than manual measurement. A previous study demonstrated that automatic calculating CAD-RADS score using structured reporting platform might play an important role in improving data quality and supporting standardization of clinical decision-making^31^. However, CAD-RADS category determined by the reporting platform was based on the data provided by the readers, although that remained hidden to the readers. DL algorithms have been widely used in the optimization of CCTA information extraction. For example, Kang et al*.* found a two-step DL algorithm based on CCTA had an high accuracy of 94% for detection of non-obstructive and obstructive CAD^12^. Furthermore, fractional flow reserve (FFR_CT_) computed by DL from CCTA features showed incremental predictive value for the risk of future adverse events^32–34^.

Several limitations of the present study should be acknowledged. First, this was a retrospective analysis of a relatively small sample size from a single center. Due to the small sample size of positive patients, we were unable to analyse the diagnostic performance of BAC with stratification by symptoms or CKD. Prospective multicentre studies of large samples will be need to enhance the application of CAD-RADS among cardiologists and radiologists. Second, in order to restrict our analysis to patients without previously known CAD, we did not include CAD-RADS modifiers to describe patients with stents (modifier S), vulnerable plaque features (modifier V), or grafts (modifier G). Further studies should include more patients in the training set and testing of DL to improve the CAD-RADS classification scheme. Finally, BAC was only described as absent or present in the current study; more precise BAC quantification or a semi-quantitative scale would be useful.

## Conclusion

We found a significant correlation between the severity of CAD detected by DL and the presence of BAC in women undergoing breast screening mammography. BAC, diabetes and LDL-C may be used as additional diagnostic criteria to predict the severity of CAD in this population. Further studies are warranted to evaluate whether the evaluation of BAC in women undergoing breast screening mammography translates into long-term clinical benefits.

## Materials and methods

This retrospective study was approved by the Central Hospital of Wuhan, Tongji Medical College, Huazhong University of Science and Technology. We confirmed that all methods were performed in accordance with the related guidelines and the principles of the Declaration of Helsinki. This study was approved by the ethics committee of the Central Hospital of Wuhan, Tongji Medical College, Huazhong University of Science and Technology. All participants gave written informed consent.

### Study population

**Figure 1:**
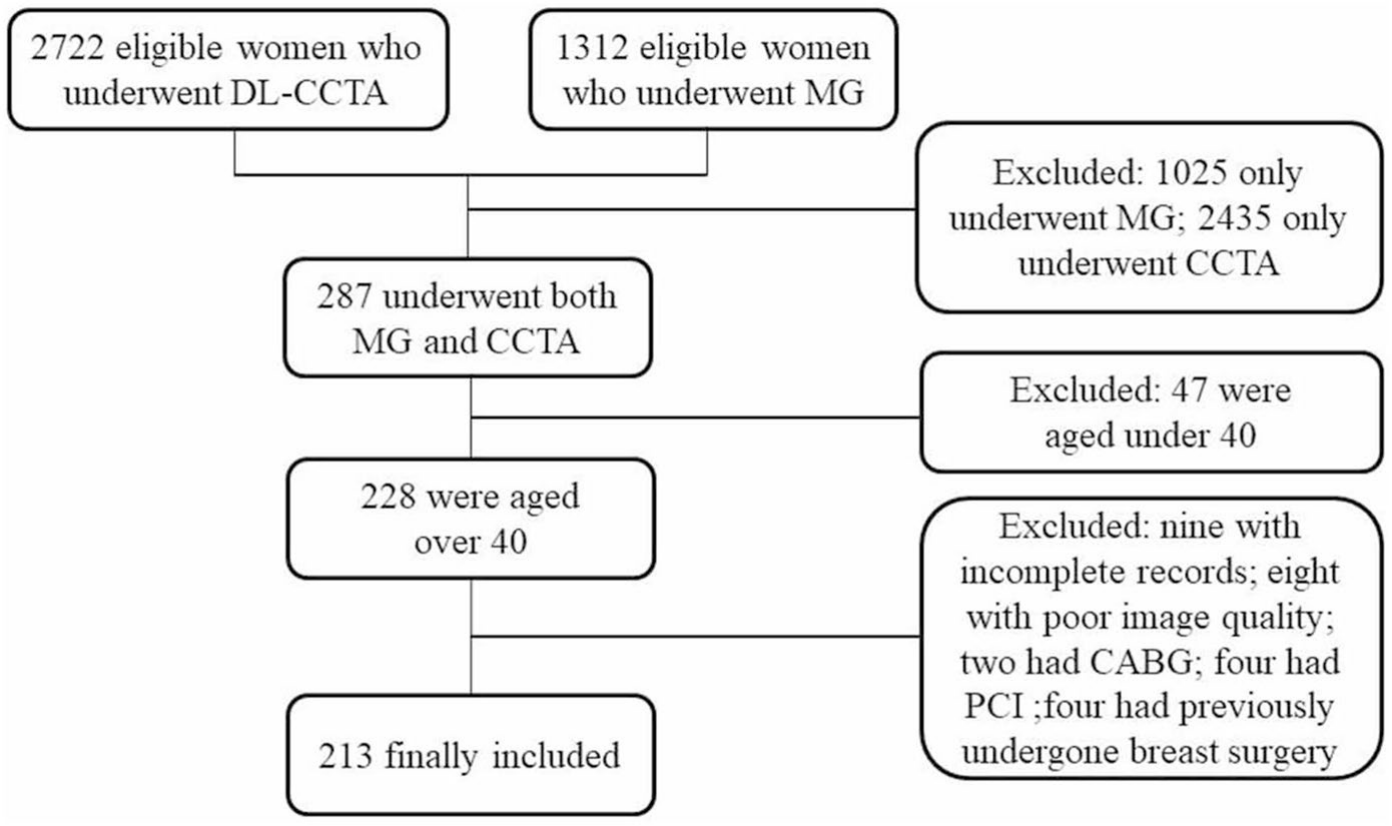
Flow chart showing inclusion of patients in the present study. *DL-CCTA* deep learning coronary computed tomography angiography, *MG* mammography, *CABG* coronary artery bypass graft, *PCI* percutaneous coronary intervention.

The institution’s Picture Archive and Communication System (PASC) were searched for female patients who were underwent both digital screening mammogram examination and DL-CCTA with an interval of less than 30 days from July 2018 to April 2019. Patients aged less than 40 were excluded, and patients with incomplete records, poor image quality, CABG, PCI and breast surgery history were also excluded. At last, a total of 213 women were included for analysis (Fig. 1). Demographic, medical history and biochemical indicators were collected and analyzed.

### Mammograms and CCTA images acquisition and analysis

Screening mammograms were performed with standard 2-view (craniocaudal and mediolateral oblique) using a full-field digital mammography system (Planmed Nuance, Planmed Oy, Helsinki, Finland). Retrospective review of the mammograms was performed by a breast radiologist with 10 years of experience, which was blinded to the clinical information and CCTA results. BAC was defined as vascular calcium deposition which was often observed as radio-opaque parallel or tubular tracks in the artery in one or both breasts^35^. In the present study, the evaluation of BAC was categorized as “0, absent” or “1, present”.

Multidetector row CT imaging was performed with dual-source CT scanner (Somatom Definition, Siemens Medical Solutions, Forchheim, Germany) which has been reported previously^36^. Heart rate control (HR ≥ 65 beats/min) was performed with beta-blockers before the scan. Scanning parameters were as following: Detector collimation 128 × 2 × 6 mm, tube voltage 120 kV, tube current 280 mAs. For contrast enhancement, 60–80 mL of iopromide (370mgI/mL, Bayer Schering Pharma, Germany) followed by 30–40 mL of pure saline with a flow rate of 4–5 mL/s. The iodine contrast agent was automatically triggered into descending aorta of 100 HU threshold units. Then the scanning was performed during an inspiratory breath hold of 8 to 14 s after delay of 2 s. The reconstruction images were automatic send to a workstation (CoronaryDoc, Shukun technology, Beijing, China) equipped with coronary analysis software tool (Computer Aided Diagnosis of Coronary Artery, Shukun technology, Beijing, China).

### DL model

#### Dataset

A total of consecutive 2000 coronary CT angiography (CCTA) examinations were included in this study. Exclusion criteria included PCI or CABG surgery, poor quality images. In the model training process, the data were randomly spitted into three sets (the training set, validation set and test set) with a 3:1:1 ratio. The training set, validation set and test set were used for training model, tuning model and evaluating model performance respectively. This diagnostic performance of this system has been validated and reported in a recent published work^37^.

#### Image labeling

Before training, the aorta, coronary artery and plaques were labeled on each image by a multi-layer manually annotation system consisting of multiple layers of trained graders. The first layer of graders is comprised of radiologists who had knowledge of medical imaging and coronary anatomy. The second layer of graders is comprised of radiologists with more than three years of work experience in radiology, which is a preliminary inspection of the accuracy of the label. The third and final layer of graders was consistence of experienced experts with over five years of work experience who verify the correctness of label of each image.

#### Auto coronary segmentation and stenosis detection

The process of our proposed Deep Convolutional Neural Network mainly contained two steps:

(1) Coronary tree segmentation. In this study, we adopted an improved 3-dimensional(3D) U-Net architecture added a Bottle-Neck design for segmentation coronary arteries and aorta, then a Growing Iterative Prediction Network (GIPN) model was developed to solve the problem of vascular segmentation fracture, final the full coronary tree segmentation was obtained. The original 3D U-net architecture has four layers for encoder and decoder respectively, to improve the architecture of 3D U-net, we added a bottle-neck design between each two layers of 3D U-net, the bottle-neck design used 1 × 1, 3 × 3 and 1 × 1 convolutions. The improved 3D U-Net architecture totally had 33 layers. The GIPN model used a crop size of 64 × 64 × 64 for fracture sites of vascular segmentation and applied 3D U-net architecture for repairing the fractures.

(2) Stenosis detection. Based on coronary tree segmentation, multiple planner reformat (MPR), curve plannar reformat (CPR), maximum intensity projection (MIP) and volume rendering (VR) images were reconstructed. To detect stenosis, we developed a 3D segmentation neural network and a one-dimensional sequence checking hybrid technique. Firstly, a 3D segmentation neural network was applied to MRP and CPR images to detect stenosis, and then a one-dimensional sequence checking algorithm was used to reduce false positive results.

**Figure 2:**
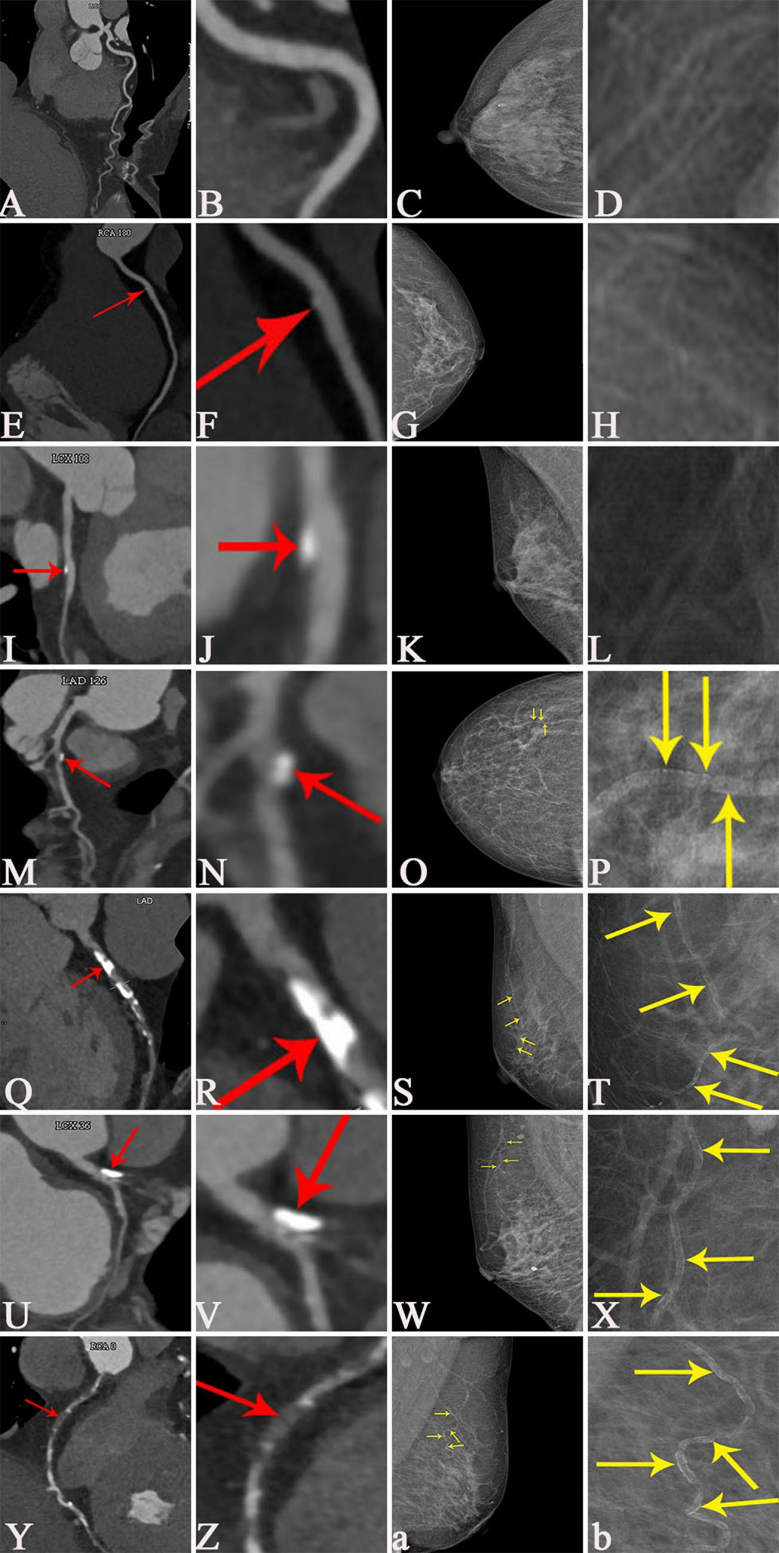
Patients’ of CAD-RADS grades evaluated by DL-based CCTA and BAC detected on breast mammograms. (**A**–**D**): Patient with CAD-RADS grade 0 (**A**, **B**) with absence of BAC (**C**, **D**). (**E**–**H**): Patient with CAD-RADS grade 1 (**E**, **F**) with absence of BAC (**G**, **H**). (**I**–**L**): Patient with CAD-RADS grade 2 (**I**, **J**) with absence of BAC (**K**, **L**). (**M**–**P**): Patient with CAD-RADS grade 3 (**M**, **N**) with presence of BAC (**O**, **P**). (**Q**–**T**): Patient with CAD-RADS grade 4A (**Q**, **R**) with presence of BAC (**S**, **T**). (**U**–**X**): Patient with CAD-RADS grade 4B (**U**, **V**) with presence of BAC (**W**, **X**). (**Y**–**b**): Patient with CAD-RADS grade 5 (**Y**, **Z**) with presence of BAC (**a**, **b**). Red arrows indicate coronary stenosis or occlusion and yellow arrows indicate BAC.

Last, the structured report was showed based the model. The CAD-RADS category was shown based on the structured report (Fig. 2).

### Statistical analysis

Continuous variables were presented as mean ± SD, and categorical variables were presented as frequencies and percentages. Quantitative data were compared using Student’s t test, chi-square test, and Fisher exact test, as appropriate. Univariate logistic analysis was performed to examine the effects of various characteristics on CAD-RADS ≥ 3. Multivariate logistic analysis were performed to evaluate whether BAC and clinical variables maintained independent associations with CAD-RADS ≥ 3 with adjusted variables using the enter method. Results were shown as odds ratio (OR) and corresponding 95% CI. A 2-sided of *P* < 0.05 was considered as statistically significant. All statistical analysis was performed using SPSS version 13 (SPSS, Inc., Chicago, IL).

## Data Availability

The datasets generated during and analyzed during the current study are available from the corresponding author on reasonable request.
